# Evaluation of Nitrogen-Corrected Apparent Metabolizable Energy and Standardized Ileal Amino Acid Digestibility of Different Sources of Rice and Rice Milling Byproducts in Broilers

**DOI:** 10.3390/ani11071894

**Published:** 2021-06-25

**Authors:** Kun Xie, Xi He, De-Xing Hou, Bingkun Zhang, Zehe Song

**Affiliations:** 1 College of Animal Science and Technology, Hunan Engineering Research Center for Poultry Safety, Hunan Agricultural University, Changsha 410128, China; k3591458@kadai.jp (K.X.); hexi111@126.com (X.H.); 2 Course of Biological Science and Technology, United Graduate School of Agricultural Sciences, Kagoshima University, Kagoshima 890-0056, Japan; k8469751@kadai.jp; 3 Hunan Co-Innovation Center of Animal Production Safety, Changsha 410128, China; 4 Department of Biochemical Science and Technology, Faculty of Agriculture, Kagoshima University, Kagoshima 890-0056, Japan; 5 State Key Laboratory of Animal Nutrition, College of Animal Science and Technology, China Agricultural University, Beijing 100083, China; bingkunzhang@126.com

**Keywords:** rice, rice byproducts, broiler chicken, nitrogen-corrected apparent metabolized energy, standardized ileal amino acid digestibility, prediction regression equation

## Abstract

**Simple Summary:**

Rice is the major cereal plant worldwide; the rice processing procedure has produced several rice byproducts that are not for human consumption but are usually used as a feed ingredient for broilers. However, due to the variation of geographic and processing methods, the quality of rice and rice byproducts is merely coincident. Thus, analysis of the chemical composition and evaluation of nutrition digestibility of rice and its byproducts in broilers and establishing the regression equation is vitally important in diet formulation and resource efficiency. Based on the above information, this study examined the differences in the chemical composition of rice, broken rice, and full-fat rice bran from the different major production areas of China, evaluated the nitrogen-corrected apparent metabolizable energy and standardized ileal amino acid digestibility in broilers by nitrogen-free diet method, established a regression equation based on partial correlation assay, and provided novel information in the diet formulation of rice, broken rice, and full-fat rice bran in broilers.

**Abstract:**

Rice, broken rice (BR), and full-fat rice bran (FFRB) from six different origins were analyzed for their chemical composition, nitrogen-corrected apparent metabolized energy (AMEn), and standardized amino acid digestibility (SIAAD) in 14-day-old and 28-day-old Arbor Acres broilers. Results showed broilers fed with rice and BR had a similar AMEn regardless of the rice and BR having different CP, EE, NDF, ADF, and ash content. FFRB containing significantly different CP, EE, NDF, ADFm and starch presented variable AMEn (*p* < 0.05), suggesting that starch content in rice and its byproducts contributed most to the AMEn of broilers. The regression equation of AMEn = 14.312 − (0.198 × NDF) and AMEn = 6.491 + (0.103 × Starch) were feasible to integrally predict AMEn of broilers fed to rice and its byproducts. Moreover, 28-day-old broilers had higher SIAAD than 14-day-old ones. The SIAAD of rice were higher than BR and FFRB except for Met, Cys, Thr, and Tyr in 14-day-old broilers (*p* < 0.05), and the SIAAD of His, Asp, and Ser in BR were higher than FFRB (*p* < 0.05). In 28-day-old broilers, the SIAAD of Leu, Trp, Asp, Gly, and Pro of rice were still higher than BR and FFRB (*p* < 0.05), but BR and FFRB had no significant differences (*p* > 0.05). The regression equations to estimate the SIAAD of Thr, Lys, and Met were: Met = 81.46 + (0.578 × CP), Thr = 0.863 + (6.311 × CP), and Trp = 102.883 − (1.77 × CP), indicating that CP content in rice and its byproducts was likely a major factor for prediction of SIAAD.

## 1. Introduction

Rice is the major cereal plant worldwide, approximately 480 million metric tons of milled rice are produced annually in the world [1]. Rice byproducts are generated in the milling processing that cover a broad array of methods to make rice cereal suitable for consumption. In rice processing, about 30% rice byproducts generated. Broken rice (BR) is one of the byproduct during the millings and rice bran is the brown layer of dehulled rice and includes several sub-layers within the pericarp and aleurone layers [2,3]. Rice byproducts are usually not for human consumption but could be used as a feed ingredient for livestock [4,5]. Compared with corn and wheat, rice and its byproducts are available for a lower price and valuable nutrition and could be partially substituted in animal feed. However, the geographic and processing methods vary greatly in rice production. Geographic environmental elements including air temperature, atmospheric carbon dioxide, light, water, and soil nutrients, that directly or indirectly impact rice nutrition content, especially starch quality and structure, which in turn inevitably determines rice milling and functional performance [6,7]. On the other hand, due to there being three milling systems used, one-step, two-step, and multi-stage milling, the different milling systems also significantly affect rice nutritional quality, resulting in macro and micronutrient content variation [8,9]. Thus, rice and its byproducts produced from different rice production areas and rice milling conditions may differ in chemical composition [10,11], which limits the usage of rice and rice byproducts in precise feed formulation.

Dietary energy sources represent a significant cost in poultry diets [12]. Corn is a major energy feedstuff, and energy is a costly dietary component [13]. Prices for energy-providing ingredients increased over 250% from 2006 to 2008, due to a portion of the corn and oil supply being diverted away from poultry agriculture for the production of ethanol and biodiesel [12,14]. Consequently, global demand and price for corn has rapidly increased. This trend forced producers to find the replicable feedstuffs that could be used as an alternative ingredient for corn in poultry diets.

Diet formulation is a kind of important combinatorial optimization problem, and it is vital to formulate the ingredients based on ileal digestible amino acids. Metabolized energy and amino acid digestibility of broilers are key aspects to assessing the energy utilization and protein quality of feed ingredients. Both the apparent ileal amino acid digestibility (AIAAD) or standardized ileal amino acid digestibility (SIAAD) are accepted in diet research [15]. However, AIAAD should not applied in practical diet formulation because this value ignores basal ileal amino acid outflow. SIAAD is a better predictor of the availability of the dietary amino acids and is more recommended in practical diet formulation [16,17].

Based on the above information, the objective of this study was to determine the chemical composition of rice, BR, and full-fat rice bran (FFRB) from six different origins in China, evaluate nitrogen-corrected apparent metabolized energy (AMEn) and standardized ileal amino acid digestibility (SIAAD) in 14-day-old and 28-day-old broilers, investigate the effect of different origins to chemical composition, and further to the SIAAD and AMEn of broilers feed with rice and its byproducts, establish prediction equations of AMEn and SIAAD for diet formulation in broilers.

## 2. Materials and Methods

The Hunan Agricultural University Animal Ethics Committee (Changsha, China) reviewed and approved all experimental protocols.

### 2.1. Rice and Rice Byproducts Collection

Six samples of each rice, BR, or FFRB were sourced from the main production areas of China, which were Hubei (rice1, BR1, and FFRB1), Hunan (rice2, BR2, and FFRB2), Anhui (rice3, BR3, and FFRB3), Heilongjiang (rice4, BR4, and FFRB4), Jiangsu (rice5, BR5, and FFRB5), Jiangxi province (rice6, BR6, and FFRB6), accounting for 60% of the yield of rice in China. Rice is grown in almost every province in China except for Qinghai. Three-quarters of the rice area is planted with indica rice varieties and the rest with japonica rice varieties. Indica rice varieties are generally grown in the south and japonicas in the north [18]. Rice growing conditions in China vary because of topography and weather, but the crop is basically irrigated. In southeastern China, high temperature and adequate rainfall make an ideal environment for rice during a long growth period, and many areas grow two crops of rice per year. In the Yangtze River Valley, much of the land is planted with a rice–wheat rotation. In northeastern China, low temperature, a short growth period, little rainfall, and a lack of water limit the rice area. The varieties grown in this area are typically japonica and were considered to be of higher quality than the rice grown in other areas. Some scattered rice areas are found in arid and semiarid regions of northwestern China. The samples numbered 1, 2, 5, 6 in this study were located in the south of China, number 4 was in the north, and number 3 was located between the south and the north of China.

### 2.2. Diet Preparation

Experimental diet formulations are presented in Table 1. A nitrogen-free diet (NFD) was used to estimate basal endogenous amino acid losses and standardized SIAAD from AIAAD. NFD was formulated according to a previous report [19], Na+, K+, and Cl− additions in NFD aimed to minimize variations in EAA losses [19]. All the diets were made into mash and fed to broilers.

### 2.3. Animal Management

A total of 1520 0-day-old Arbor Acres chickens were housed in an environmentally controlled room and the temperature was set at 34 °C on the first day, then descended 2 °C per week. Birds were acclimatized with a basal diet designed based on NRC (1994) and free access to water and feed. At 7 days old, 912 birds were allotted to experimental diets by using a completely randomized design with 19 treatments, 8 replicate cages of 6 birds per cage. The rest of the birds continued to be fed with a basal diet. Then, at 21 days old, the remaining 608 birds were assigned to experimental diets by using the same method with 19 treatments, 8 replicates of 4 birds per cage. Experimental diets were fed during each phase of growth for 7 d, which was composed of 4 d of adaptation and 3 d of excreta collection. During the excreta collection period, wax paper was placed under each cage. To avoid fermentation and nitrogen losses, 6 N HCl was sprinkled onto the excreta after collection. The wax paper for each cage was changed every day and collected excreta were stored at −20 °C in a freezer. After collection, all the excreta were pooled per cage and dried in a forced air oven at 65 °C, then excreta were grounded to pass through a 1-mm screen mesh and stored at −20 °C for AME measurement. At the end of the 7 d period, the 14-day-old and 28-day-old birds were euthanized to collect ileal digesta for SIAAD determination. The ileum was defined as the portion of the small intestine extending from Meckel’s diverticulum to a point of 40 mm proximal to the ileocaecal junction. Ileal digesta were freeze-dried and stored at −80 °C for AIAAD measurement.

### 2.4. Chemical Composition and Apparent Metabolized Energy Assay

Ingredients were ground to pass through a 1-mm screen mesh, and measured for crude protein (method 984.13; AOAC,2006), crude fat (method 920.39; AOAC,2006), calcium (method 968.08; AOAC,2006), and total phosphorus (method 964.06; AOAC,2006). Neutral detergent fiber (NDF) and acid detergent fiber (ADF) were analyzed by a fiber analyzer (ANKOM A200i; Beijing, China.). Starch was determined by a starch quantities kit (Sigma-Aldrich; Shanghai, China) according to the manuscript. The gross energy (GE) of diets and fecal samples were determined with Oxygen Bomb Automatic Calorimeter (HXR-6000, Hunan Huaxing Energy Sources Instrument Co. Ltd., Changsha, China). AME was calculated according to the following equation:

$$\mathrm{AME}=E_{\mathrm{diet}\;\mathrm{per}\;\mathrm{gram}}-\frac{{{\mathrm{TiO}}_{2}}_{\mathrm{per}\;\mathrm{gram}\;\mathrm{diet}}}{{{\mathrm{TiO}}_{2}}_{\mathrm{per}\;\mathrm{gram}\;\mathrm{excreta}}}\times E_{\mathrm{excreta}\;\mathrm{per}\;\mathrm{gram}}$$

Nitrogen-corrected AME (AMEn) was calculated by correction to zero nitrogen retention according to Hill and Anderson (1958) [20], which in brief,

$$N=N_{\mathrm{per}\;\mathrm{gram}\;\mathrm{diet}}-\left(N_{\mathrm{per}\;\mathrm{gram}\;\mathrm{feces}}\times \frac{{{\mathrm{TiO}}_{2}}_{\mathrm{per}\;\mathrm{gram}\;\mathrm{diet}}}{{{\mathrm{TiO}}_{2}}_{\mathrm{per}\;\mathrm{gram}\;\mathrm{feces}}}\right)$$

$$\mathrm{AMEn}=\mathrm{AME}=E_{\mathrm{diet}\;\mathrm{per}\;\mathrm{gram}}-\frac{{{\mathrm{TiO}}_{2}}_{\mathrm{per}\;\mathrm{gram}\;\mathrm{diet}}}{{{\mathrm{TiO}}_{2}}_{\mathrm{per}\;\mathrm{gram}\;\mathrm{excreta}}}\times E_{\mathrm{excreta}\;\mathrm{per}\;\mathrm{gram}}-8.22\times N$$

N represents nitrogen retention per gram of diet dry matter. To analyze the AA content in diets and ileal digesta, ileal digesta and diets were hydrolyzed in 6N HCl for 24 h at 110 °C under N atmosphere. The AA in the hydrolysate was subsequently determined by HPLC which equipped the amino acid analysis column (4.6 × 100 mm, 2.7 µm, Agilent; Shanghai, China).

### 2.5. Amino Acid Digestibility

SIAAD was corrected from AIAAD by eliminating the endogenous amino acid lost. Basal ileal endogenous amino acid losses were measured by NFD, and SIAAD values were calculated by the indicator TiO_2_ (5 g/kg) added in each diet. The SIAAD was calculated according to the formulation reported by Stein (2007) and Iyayi, E. A. (2021) [16,21]:

$$\mathrm{AIAAD}=\left[1-\left(\frac{{\mathrm{AA}}_{\mathrm{digesta}}}{{\mathrm{AA}}_{\mathrm{diet}}}\right)\times \left(\frac{{{\mathrm{TiO}}_{2}}_{\mathrm{diet}}}{{{\mathrm{TiO}}_{2}}_{\mathrm{digesta}}}\right)\right]\times 100$$

$${\mathrm{IAA}}_{\mathrm{endogenous}}={\mathrm{AA}}_{\mathrm{NFD}\;\mathrm{digesta}}\times \left(\frac{{{\mathrm{TiO}}_{2}}_{\mathrm{diet}}}{{{\mathrm{TiO}}_{2}}_{\mathrm{NFD}\;\mathrm{digesta}}}\right)$$

$$\mathrm{SIAAD}=\mathrm{AIAAD}+\left[\left(\frac{{\mathrm{IAA}}_{\mathrm{endogenous}}}{{\mathrm{AA}}_{\mathrm{diet}}}\right)\times 100\right]$$

### 2.6. Statistical Analysis

Results of the chemical composition of ingredients were expressed as mean ± SD; AME, AMEn, and SIAAD of broilers fed rice, BR, and FFRB from different origins were expressed as mean ± SEM. The significant differences of chemical composition were analyzed by one-way ANOVA, followed by Tukey’s post hoc tests with the SPSS statistical program (version 19.0, IBM Corp., Armonk, NY, USA.). Statistical significance was declared at *p* < 0.05. Significant differences of AME, AMEn, and SIAAD between origins, ages, and their interaction were analyzed by two-way ANOVA, followed by Tukey’s post hoc tests in SPSS statistical program, statistical significance was set at *p* < 0.05. The correlation tests between chemical composition and AMEn, SIAAD of broilers were performed by a partial correlation test, different ingredients and age were set as controlling coefficients. Regression of the test ingredients associated with AMEn and SIAAD were conducted using stepwise liner regression procedure in SPSS statistical program.

## 3. Results

### 3.1. Chemical Composition

The chemical composition of rice, BR, and FFRB are shown in Table 2, Table 3 and Table 4, respectively. The concentration of EE, NDF, and ADF showed significant differences in rice and BR from different sources (*p* < 0.05). In contrast, GE, DM, CP, total starch, ash, calcium, and total phosphorus showed no significant difference in rice and BR of different origins (*p* > 0.05). Thr, Met, Asp, and Ser content have significant differences in rice and broken rice from different sources (*p* < 0.05). Rice and BR have a similar chemical composition except that rice has a slightly lower CP, NDF, ash, Met, Ser, and Tyr content (Table 5, *p* < 0.05). The chemical composition of different origin FFRB presented massive significant differences in GE, CP, EE, ADF, NDF, and total starch (*p* < 0.05); among them, ADF, NDF, and total starch showed the most variability (*p* < 0.01). DM, ash, calcium, and total phosphorus in FFRB from different origins did not present differences (*p* > 0.05). Amino acids of Thr, Val, Lys, Asp, Ser, Glu, Pro, and Gly concentration showed significant differences (*p* < 0.05).

### 3.2. AME and AMEn Assay

The AME and AMEn of broilers fed with each experimental diet are presented in Table 6. Regardless of rice and BR, there were no differences in AME and AMEn between the different sources fed to the broilers at both ages (*p* > 0.05), and neither ages nor ages interacting with origins showed any significant differences (*p* > 0.05). However, broilers fed with FFRB from different origins presented significant differences in AME and AMEn (*p* < 0.05). FFRB4- and FFRB5-fed broilers presented the lowest AMEn, and among all the FFRB groups, FFRB4 had the lowest EE and total starch content, FFRB5 had the highest NDF and ADF concentration. Moreover, broilers fed with FFRB showed no statistical differences in the AME and AMEn in ages (*p* > 0.05). Pearson’s correlation assay was used to analyze the association between AMEn and chemical composition of experimental diets. As shown in Supplement Table S1, DM and total starch content were positively correlated with AMEn (*p* < 0.05), the other chemical compositions wereall negatively correlated with AMEn (*p* < 0.05).

### 3.3. Amino Acid Digestibility

The evaluation of endogenous AA loss enabled us to establish the SIAAD of feed ingredients. As shown in Table 7, the average SIAAD of Trp in 14-day-old broilers was the highest (89.60%), Trp, Phe, and His in 28-day-old broilers were the highest (92.23%, 92.18%, and 92.47%, respectively). The SIAAD of Arg and Ala of broilers fed with rice showed significant original differences (*p* < 0.05), and the SIAAD of His, Ile, Met, Phe, Trp, Cys, Glu, Gly, and Ser had significant age differences in broilers fed rice (*p* < 0.05). The SIAAD of Met had significant interaction between origin and age (*p* < 0.05). In Table 8, the highest SIAAD of BR were Trp and Phe (88.26% and 88.12%) in 14-day-old broilers, and the SIAAD of Glu, Phe (90.03%, 90.15%) were the highest in 28-day-old broilers. The SIAAD of Leu and Ala showed significant original differences in broilers fed with BR (*p* < 0.05), and His, Leu, Val, Cys, and Glu had marked differences in ages (*p* < 0.05). The interaction between ages and origins had no significant differences (*p* > 0.05). As shown in Table 9, the average SIAAD of Met of FFRB in 14-day-old broilers was the highest (87.42%), and Met and Glu in 28-day-old broilers were the highest (90.58% and 90.61%). The SIAAD of Leu, Thr, Trp, Val, and Ala in broilers fed with FFRB showed statistically significant differences (*p* < 0.05). Arg, His, Ile, Leu, Lys, Met, Phe, Ala, Glu, Gly, and Tyr of SIAAD had significant differences in ages (*p* < 0.05). Moreover, the SIAAD of Leu, Met, and Ala had significant interactions between age and origin (*p* < 0.05). Overall, the SIAAD of rice, BR, and FFRB increased with age after standardization, which is consistent with a previous report [17]. The significant interaction of the SIAAD of Met in rice, and Leu, Met, and Ala in FFRB suggests that with the development of digestive tract of broilers, the effect on AA digestibility may gradually be revealed due to the different chemical composition of ingredients from different origins. The SIAAD of rice was significantly higher than BR and FFRB except for Met, Cys, Thr, and Tyr in 14-day-old broilers (Table 10, *p* < 0.05), and the SIAAD of His, Asp, and Ser of BR were higher than FFRB in 14-day-old broilers (*p* < 0.05), but the SIAAD of Arg was lower (*p* < 0.05). In 28-day-old broilers, the SIAAD of Leu, Trp, Asp, Gly, and Pro of rice was still higher than BR and FFRB (*p* < 0.05). The SIAAD of BR and FFRB had no significant differences (*p* > 0.05).

### 3.4. Regression Equation

Liner regression equation of AMEn and SIAAD of broilers fed rice and its byproducts were established (Table 11) by using the stepwise method based on partial correlation assay, the age and different origins were set as controlling factors. For simplicity and practicality, the SIAAD of Thr, Lys, Met, and Trp, which are critical in practical diet formulation [22,23], were presented in the regression equation. As shown in Table 11, the AMEn of the broilers had the best correlation to the chemical composition of NDF and starch. According to the regression analysis, AMEn elevated with the concentration of starch increasing, but reduced with NDF increasing (*p* < 0.05). Furthermore, the SIAAD of Met increased with the protein concentration increasing (*p* < 0.05), however, Trp decreased with protein concentration increasing (*p* < 0.05). In particular, the precise of regression equation of the SIAAD of Lys was insufficient with only one chemical composition coefficient. The best fit equation was X = 62.00 + (0.938STARCH − 0.021 × NDF).

## 4. Discussion

The physical and chemical composition of rice and its byproducts may depend on geographic factors, the treatment of milling, and the fractionation method [3]. During the milling process, microbial activity is involved which produces lipase hydrolysates [24]. This turns the oil in BR and FFRB into glycerol and free fatty acids, which gives the product its rancid smell and bitter taste that renders the FFRB unsuitable for human consumption but acceptable as a feed ingredient for broilers. In this study, rice, BR, and FFRB from different origins were examined for their chemical composition. Rice and BR have different CP, EE, NDF, ADF, and ash content. FFRB contained significantly different CP, EE, NDF, ADF, and starch. Furthermore, the concentrations of total starch and crude protein of rice and BR were lower than suggested in a previous report [25]; NDF and EE were greater than in the previous study [25]. Several studies also suggested that FFRB has a variable chemical composition [26,27,28]. The CP content of different sources of FFRB ranged from 13.6% to 21.0%, and crude fat and NDF ranged from 4.1% to 24.4% and 2.1% to 34.3%, respectively [29]. The average CP content of FFRB in our study was 13.15 ± 0.74%, EE was 13.43 ± 1.63%, and NDF was 27.30 ± 5.91%, which had a lower CP but a higher NDF than previous study [27,29]. These data indicated that the quality of FFRB is merely coincident and limited its application in precise diet formulation. However, FFRB contains considerable nutrition, and dietary exogenous enzymes supplementation showed significant improvement in the broiler availability of FFRB [30,31,32]. Therefore, it is necessary to dynamically evaluate the nutritional value of rice bran through the regression equation.

Rice and BR in animal feedstuff are responsible for energy providing and could be directly used in broilers’ diet due to their compromised anti-nutrition factors [27,33]. FFRB, as a potential substitution for maize or wheat [32,34], has a considerable crude protein and fat content, but high NDF and ADF content may limit the utilization of FFRB in animal feed [4]. In the present study, the correlation assay between chemical composition and AMEn revealed that DM and total starch are the only two factors that had a positive association with AMEn, which may indicate that starch is principally responsible for providing metabolized energy in rice and its byproducts when fed to broilers. The total starch content of rice and BR ranged from 66.62% to 70.68% and 66.28% to 69.14%, respectively. The digestion of starch generally occurred in the small intestine, with the candidate of α-amylase. Furthermore, α-amylase inhibitors have been found in wheat, rye, triticale, and sorghum, but not in rice, barley, and maize [35]. In the present study, rice and BR with different CP, EE, NDF, ADF, and ash content did not present differences in AMEn at 14 days old or 28 days old. However, variation of AMEn in FFRB fed broilers was observed, The most significantly different chemical compositions in FFRB were NDF, ADF, and starch; NDF ranged from 20.57% to 34.83%, ADF range from 7.37% to 12.54%, and starch ranged from 18.86% to 27.57%. The different starch levels may contribute to variable AMEn, and the relative high concentration of NDF and ADF may limit the AMEn in broilers [34,36,37]. Thus, establishing the regression equation is vitally important for employing FFRB. The correlation assay revealed that NDF and ADF were most correlated to AMEn. Moreover, the regression equation of AMEn showed that NDF content was best fit for prediction, and NDF as a relatively stable composition has been used to establish the prediction equation of ME in swine fed to DDGS [38,39]. Thus, this study suggested that NDF content in rice and its byproducts may also be possible to use to predict the AMEn in broilers. On the other hand, the correlation assay showed that EE represented a significant negative correlation to AMEn. Several studies concluded that EE contributed to providing GE in corn-DDGS, but its apparent total tract digestibility was quite variable among corn-DDGS sources in both experiments, indicating that EE was not a primary factor for predicting DE or ME in growing pigs [40]. The data in swine fed different sources DDGS also suggested that the fiber component of DDGS has a greater impact on ME than does EE content [38]. This indicated that although EE is rich in FFRB and contributed to the gross energy of diet, it may not be responsible for the AMEn of broilers fed the FFRB diet. Because the EE in FFRB could easily become rancid, it is suggested that it may not be the ideal input in energy prediction equations for FFRB-fed broilers.

Feed formulations based on digestible amino acids could improve the precise of nutritive value of feed ingredients and get closer to the true utilization of broilers [36]. Accumulated studies have shown that BR has a similar AA digestibility to corn in broilers [37]. A study reported that feeding rice instead of corn increased nutrients digestibility and growth performance in pigs [41,42]. BR has a higher Met, Ser, and Tyr content than rice in our study. These differences might due to the polishing process which removed the aleurone layer that is rich in amino acid [3]. Though rice and BR are not protein-rich ingredients, they have a higher digestibility and fewer anti-nutrition factors than other cereal plants [43]. FFRB including an aleurone layer and germ together has a higher protein content, which could be around 15% in FFRB and 20% in defatted rice bran [10]. In addition, rice bran contains various biological proteins, such as rice bran lipase and catalase, that have health benefits [44,45]. FRRB in this study contained 13.15 ± 0.74% CP. However, the fiber content in FFRB was variable [29], and may have a negative effect on AA digestibility [46,47]. A diet formulated based on amino acid of both full-fat or defatted rice bran will lead to variable nutrition value and finally result in the poor performance of broilers. Consistent with our study, amino acid composition in FFRB showed that Thr, Val, Lys, Asp, Ser, Glu, Pro, and Gly had significant different concentration. Thus, evaluating the SIAAD of broilers fed with FFRB and establishing a regression equation to provide the baseline for practical production is critically important. In our study, the best fit SIAAD of Met, Thr, and Trp equations were Met = 81.46 + (0.578 × CP), Thr = 0.863 + (6.311 × CP), and Trp = 102.883 − (1.77 × CP), respectively. Due to AIAAD increases corresponding to dietary AA intake [15], and endogenous AA losses remaining relatively stable, the CP content of the feed ingredients was likely a major factor for the prediction of SIAAD [15,48]. However, the precision of prediction equations for the SIAAD of Lys with CP content was insufficient. The best fit equation was X = 62.00 + (0.938STARCH − 0.021 × NDF), suggesting that starch and NDF content may affect AA digestibility, and could be considered as an alternative factor for the prediction of SIAAD.

In summary, our study demonstrated that broilers fed with rice and BR had a similar AMEn regardless of the fact that rice and BR had different CP, EE, NDF, ADF, and ash content. FFRB containing significantly different CP, EE, NDF, ADF, and starch presented variable AMEn, suggesting that starch content in rice and its byproducts contributed most to the AMEn of the broilers. The regression equations of AMEn = 14.312 − (0.198 × NDF) and AMEn = 6.491 + (0.103 × Starch) were feasible to integrally predict the AMEn of broilers fed with rice and its byproducts. Moreover, 28-day-old broilers had a higher SIAAD than 14-day-old. The SIAAD of rice was higher than that of BR and FFRB except for Met, Cys, Thr, and Tyr in 14-day-old broilers, and the SIAAD of His, Asp, and Ser of BR were higher than FFRB. In 28-day-old broilers, the SIAAD of Leu, Trp, Asp, Gly, and Pro of rice were still higher than BR and FFRB, but BR and FFRB had no significant differences. The study provided a regression equation to estimate the SIAAD of Thr, Lys, Met, and Trp in broilers fed with rice and its byproducts, and could be considered when formulating broilers’ feed.

## Figures and Tables

**Table 1:** Experiment diet formulation (g/kg as-fed basis).

	Rice	BR	FFRB	NFD ^1^
Ingredient g/kg				
Corn starch	0	0	0	198
Dextrose	0	0	0	640
Feed ingredient	924	924	924	0
NaHCO_3_	0	0	0	7.5
KCl	0	0	0	7.5
MgO	0	0	0	2
Solkafloc	25	25	25	50
Soy oil	0	0	0	50
Monocalcium phosphate	20	20	20	19
Choline chloride	3	3	1	3
Limestone	15	15	12	13
Sodium chloride	3	3	3	0
Vitamin mineral premix ^2^	5	5	5	5
TiO_2_	5	5	5	5
Total	1000	1000	1000	1000

^1^ NFD = Nitrogen-free diet. ^2^ Provided per kilogram of diet: vitamin A, 9500 IU; vitamin D3, 62.5 μg; vitamin K3, 2.65 mg; vitamin B1, 2 mg; vitamin B2, 6 mg; vitamin B12, 0.025 mg; vitamin E, 30 IU; biotin, 0.0325mg; folic acid, 1.25 mg; pantothenic, 12 mg; niacin, 50 mg; Cu, 8 mg; Zn, 75 mg; Fe, 80 mg; Mn, 100 mg; Se, 0.15 mg; I, 0.35 mg.

**Table 2:** Analyzed nutrition and amino acid composition of different sources rice (%).

Nutrition	Rice 1	Rice 2	Rice 3	Rice 4	Rice 5	Rice 6	*p*-Value	Mean	SD
GE (MJ/kg)	17.45	17.43	17.73	18.41	18.37	17.59	0.124	17.83	0.45
Dry matter	89.09	88.13	88.20	88.63	89.47	90.58	0.204	89.02	0.92
Crude protein	7.03	6.92	6.74	6.97	7.17	7.11	0.325	7.31	0.15
Ether extract	1.19	1.41	3.22	3.79	2.35	3.33	0.012	2.55	1.08
NDF ^1^	2.38	3.74	1.17	1.83	3.02	3.34	0.044	2.58	0.97
ADF ^1^	0.71	0.67	0.71	0.21	0.45	1.21	0.031	0.66	0.33
Total Starch	66.62	70.68	69.59	66.84	69.56	69.79	0.334	68.85	1.69
Ash	0.45	0.38	0.37	0.47	0.47	0.36	0.102	0.41	0.05
Calcium	0.044	0.034	0.045	0.025	0.033	0.035	0.078	0.04	0.01
Total phosphorus	0.22	0.28	0.24	0.21	0.19	0.24	0.504	0.23	0.03
Indispensable AA									
Arg	0.44	0.46	0.38	0.54	0.46	0.52	0.305	0.46	0.06
His	0.15	0.21	0.13	0.29	0.2	0.31	0.208	0.21	0.07
Ile	0.25	0.28	0.22	0.31	0.28	0.3	0.341	0.27	0.03
Leu	0.48	0.61	0.45	0.65	0.6	0.64	0.445	0.57	0.09
Lys	0.28	0.34	0.29	0.37	0.37	0.36	0.550	0.34	0.04
Met	0.22	0.16	0.18	0.14	0.16	0.15	0.037	0.17	0.03
Phe	0.36	0.39	0.34	0.44	0.39	0.43	0.459	0.39	0.04
Thr	0.36	0.26	0.38	0.34	0.3	0.31	0.032	0.32	0.04
Trp	0.12	0.16	0.19	0.12	0.11	0.11	0.328	0.14	0.03
Val	0.3	0.38	0.3	0.44	0.4	0.42	0.259	0.37	0.06
Total	3.14	3.36	3.03	3.81	3.40	3.69	0.215	3.39	0.30
Dispensable AA									
Ala	0.36	0.43	0.33	0.47	0.44	0.46	0.187	0.41	0.06
Asp	0.63	0.64	0.57	0.71	0.69	0.71	0.021	0.66	0.06
Cys	0.18	0.11	0.17	0.17	0.13	0.14	0.683	0.15	0.03
Glu	1.27	1.45	1.2	1.55	1.41	1.56	0.150	1.41	0.15
Gly	0.31	0.33	0.29	0.37	0.34	0.37	0.124	0.33	0.03
Pro	0.39	0.58	0.39	0.45	0.43	0.36	0.162	0.43	0.08
Ser	0.36	0.38	0.35	0.42	0.37	0.41	0.013	0.38	0.03
Tyr	0.18	0.14	0.16	0.18	0.12	0.15	0.113	0.16	0.02
Total	3.5	3.95	3.29	4.15	3.80	4.02	0.009	3.78	0.33

^1^ NDF means neutral detergent fiber, ADF means acid detergent fiber. Data were presented as mean ± SD, each ingredient from the different origins were used with a stratified sampling method and tested four times as replicates.

**Table 3:** Analyzed nutrition and amino acid composition of different sources BR (%).

Nutrition	BR 1	BR 2	BR 3	BR 4	BR 5	BR 6	*p*-Value	Mean	SD
GE (MJ/kg)	17.40	17.12	17.68	17.18	18.05	17.67	0.329	17.52	0.35
Dry matter	89.02	88.38	88.55	90.20	89.78	92.68	0.269	89.77	1.59
Crude protein	7.95	7.58	7.38	7.61	7.05	7.65	0.428	7.54	0.30
Ether extract	3.95	4.79	2.27	3.19	4.00	3.37	0.016	3.60	0.86
NDF ^1^	7.48	3.27	5.02	4.02	8.84	3.35	0.008	5.33	2.32
ADF ^1^	0.79	0.66	0.86	0.43	0.56	0.71	0.041	0.67	0.16
Total Starch	69.14	66.78	68.46	66.28	68.02	69.03	0.846	67.95	1.18
Ash	0.52	0.54	0.71	0.56	0.60	0.68	0.340	0.47	0.08
Calcium	0.032	0.026	0.041	0.035	0.034	0.028	0.103	0.03	0.01
Total phosphorus	0.27	0.21	0.22	0.18	0.22	0.19	0.664	0.22	0.03
Indispensable AA									
Arg	0.55	0.7	0.61	0.45	0.52	0.4	0.402	0.54	0.11
His	0.23	0.26	0.34	0.26	0.32	0.24	0.274	0.28	0.04
Ile	0.28	0.26	0.24	0.24	0.27	0.25	0.449	0.26	0.02
Leu	0.62	0.78	0.58	0.53	0.61	0.56	0.586	0.61	0.09
Lys	0.32	0.31	0.31	0.33	0.36	0.33	0.724	0.33	0.02
Met	0.24	0.23	0.16	0.29	0.29	0.25	0.049	0.24	0.05
Phe	0.4	0.46	0.41	0.4	0.39	0.39	0.604	0.41	0.03
Thr	0.34	0.37	0.28	0.29	0.28	0.4	0.017	0.33	0.05
Trp	0.13	0.15	0.11	0.1	0.11	0.12	0.432	0.12	0.02
Val	0.39	0.45	0.41	0.4	0.39	0.4	0.341	0.41	0.02
Total	3.5	3.97	3.45	3.29	3.54	3.34	0.283	3.67	0.24
Dispensable AA									
Ala	0.42	0.52	0.45	0.43	0.48	0.43	0.246	0.46	0.04
Asp	0.66	0.66	0.65	0.79	0.72	0.72	0.028	0.7	0.05
Cys	0.13	0.14	0.21	0.12	0.19	0.16	0.899	0.16	0.04
Glu	1.73	1.77	1.55	1.44	1.52	1.45	0.198	1.58	0.14
Gly	0.32	0.36	0.37	0.39	0.43	0.4	0.132	0.38	0.04
Pro	0.32	0.36	0.33	0.47	0.35	0.4	0.213	0.37	0.06
Ser	0.38	0.46	0.41	0.52	0.56	0.6	0.017	0.49	0.09
Tyr	0.18	0.2	0.23	0.22	0.18	0.17	0.517	0.2	0.02
Total	4.01	4.33	3.99	4.26	4.24	4.17	0.042	4.17	0.14

^1^ NDF means neutral detergent fiber, ADF means acid detergent fiber. Data were presented as mean ± SD, each ingredient from the different origins were used with a stratified sampling method and tested four times as replicates.

**Table 4:** Analyzed nutrition and amino acid composition of different sources FFRB (%).

Nutrition	FFRB1	FFRB2	FFRB3	FFRB4	FFRB5	FFRB6	*p*-Value	Mean	SD
GE(MJ/kg)	19.74	20.52	18.10	18.30	18.16	20.65	0.001	19.25	1.20
Dry matter	86.13	86.33	87.2	88.01	86.14	87.29	0.671	86.85	0.77
Crude protein	13.35	13.02	11.89	13.08	13.41	14.16	0.036	13.15	0.74
Ether extract	15.35	12.22	13.7	11.28	12.83	15.17	0.012	13.43	1.63
NDF ^1^	20.57	24.56	33.31	28.51	34.83	22.03	<0.001	27.30	5.91
ADF ^1^	7.91	7.37	12.26	11.98	12.54	8.53	<0.001	10.10	2.40
Total Starch	27.06	27.57	25.25	18.86	21.23	21.63	<0.001	23.60	3.53
Ash	8.36	8.62	8.91	9.24	7.25	9.27	0.062	8.61	0.75
Calcium	0.30	0.22	0.20	0.20	0.20	0.18	0.881	0.22	0.04
Total phosphorus	2.03	2.19	1.88	1.93	2.13	1.97	0.223	2.02	0.12
Indispensable AA									
Arg	1.04	1.1	0.84	0.8	0.85	1.1	0.075	0.96	0.14
His	0.39	0.37	0.35	0.33	0.38	0.49	0.068	0.39	0.06
Ile	0.45	0.52	0.47	0.41	0.45	0.49	0.334	0.47	0.04
Leu	0.95	0.83	0.88	0.9	0.81	1.08	0.178	0.91	0.10
Lys	0.7	0.78	0.55	0.54	0.71	0.8	0.037	0.68	0.11
Met	0.23	0.23	0.25	0.27	0.21	0.29	0.533	0.25	0.03
Phe	0.54	0.55	0.56	0.61	0.54	0.64	0.745	0.57	0.04
Thr	0.69	0.74	0.77	0.67	0.59	0.79	0.012	0.71	0.07
Trp	0.16	0.22	0.16	0.22	0.2	0.23	0.328	0.20	0.03
Val	0.6	0.61	0.56	0.53	0.57	0.8	0.019	0.61	0.10
Total	5.75	5.95	5.39	5.28	5.31	6.71	0.025	5.73	0.55
Dispensable AA									
Ala	0.76	0.84	0.73	0.71	0.65	0.85	0.751	0.76	0.08
Asp	1.26	1.39	0.96	0.95	1.24	1.38	0.012	1.20	0.20
Cys	0.22	0.21	0.25	0.22	0.21	0.27	0.624	0.23	0.02
Glu	2.13	2.09	1.56	1.52	1.69	1.91	0.001	1.82	0.27
Gly	0.58	0.61	0.43	0.59	0.61	0.75	0.024	0.60	0.10
Pro	0.73	0.7	0.55	0.5	0.5	0.54	0.022	0.59	0.10
Ser	0.6	0.64	0.43	0.53	0.54	0.65	0.033	0.57	0.08
Tyr	0.32	0.32	0.28	0.25	0.25	0.32	0.342	0.29	0.03
Total	12.35	12.75	10.58	10.55	11	13.38	0.009	11.77	1.22

^1^ NDF means neutral detergent fiber, ADF means acid detergent fiber. Data were presented as mean ± SD, each ingredient from the different origins were used with a stratified sampling method and tested four times as replicates.

**Table 5:** Comparison of nutrition and amino acid of rice and BR ingredients.

Item%	Rice	BR	SEM	*p*-Value
GE (kcal/kg)	17.83	17.52	0.23	0.206
DM	89.02	89.77	0.75	0.340
Crude protein	6.99	7.54	0.14	0.003
Ether extracts	2.55	3.60	0.56	0.092
NDF ^1^	2.58	5.33	1.03	0.023
ADF ^1^	0.66	0.67	0.15	0.957
Total Starch	68.85	67.95	0.84	0.313
Ash	0.42	0.60	0.04	0.001
Calcium	0.04	0.03	0.00	0.418
Total phosphorus	0.23	0.22	0.02	0.425
Indispensable AA				
Arg	0.47	0.54	0.05	0.183
Cys	0.15	0.16	0.02	0.659
His	0.22	0.28	0.03	0.115
Ile	0.27	0.26	0.02	0.296
Leu	0.57	0.61	0.05	0.424
Lys	0.34	0.33	0.02	0.656
Met	0.17	0.24	0.02	0.008
Phe	0.39	0.41	0.02	0.404
Thr	0.33	0.33	0.03	0.953
Trp	0.14	0.12	0.02	0.348
Val	0.37	0.41	0.03	0.233
Total	3.41	3.67	0.16	0.121
Dispensable AA				
Ala	0.42	0.46	0.03	0.183
Asp	0.66	0.7	0.03	0.216
Glu	1.41	1.58	0.08	0.068
Gly	0.34	0.38	0.02	0.057
Pro	0.43	0.37	0.04	0.148
Ser	0.38	0.49	0.04	0.016
Tyr	0.16	0.2	0.01	0.013
Total	3.79	4.17	0.15	0.026

^1^ NDF means neutral detergent fiber, ADF means acid detergent fiber.

**Table 6:** Nitrogen-corrected apparent metabolized energy of different sources test ingredients in broilers (%).

Item	AME (MJ/kg)		AMEn (MJ/kg)	
	14-Day-Old	28-Day-Old	14-Day-Old	28-Day-Old
Rice 1	13.91	13.66	13.82	13.32
Rice 2	14.12	13.79	13.35	13.13
Rice 3	13.69	13.62	13.27	13.93
Rice 4	13.82	13.67	13.79	13.31
Rice 5	14.29	13.95	13.01	13.04
Rice 6	14.07	13.68	13.33	13.76
Mean	13.98	13.73	13.43	13.42
SEM	0.09	0.05	0.13	0.14
*p*-value (Origin)	0.762		0.694	
*p*-value (Age)	0.308		0.422	
*p*-value (Origin × Age)	0.894		0.920	
BR 1	14.35	14.14	13.58	13.54
BR 2	14.11	14.08	13.63	13.50
BR 3	14.03	13.99	13.37	13.38
BR 4	14.33	14.18	13.65	13.52
BR 5	14.35	14.11	13.74	13.69
BR 6	14.11	14.08	13.76	13.66
Mean	14.21	14.10	13.62	13.55
SEM	0.06	0.03	0.06	0.05
*p*-value (Origin)	0.335		0.155	
*p*-value (Age)	0.764		0.622	
*p*-value (Origin × Age)	0.853		0.775	
FFRB 1	12.35	11.92	11.99	11.57
FFRB 2	10.92	10.47	10.24	9.82
FFRB 3	9.15	8.72	7.57	7.19
FFRB 4	8.53	8.09	7.73	7.28
FFRB 5	8.27	7.81	7.23	6.81
FFRB 6	10.84	10.30	10.28	9.77
Mean	10.01	9.55	9.17	8.74
SEM	0.66	0.66	0.79	0.79
*p*-value (Origin)	0.001		0.001	
*p*-value (Age)	0.079		0.687	
*p*-value (Origin × Age)	0.749		0.837	

**Table 7:** Standardized amino acid digestibility of different sources rice in broilers (%).

Item	14-Day-Old								28-Day-Old								*p*-Value (Origin)	*p*-Value (Age)	*p*-Value (Interact)
	Rice 1	Rice 2	Rice 3	Rice 4	Rice 5	Rice 6	Mean	SEM	Rice 1	Rice 2	Rice 3	Rice 4	Rice 5	Rice 6	Mean	SEM			
Indispensable AA																			
Arg	84.00	92.45	82.62	87.12	87.08	90.47	87.29	1.15	87.79	88.75	84.02	90.38	90.98	90.60	88.75	1.00	0.048	0.450	0.805
His	87.79	90.97	84.48	89.56	88.88	88.24	88.32	0.87	92.93	94.31	88.98	92.85	93.56	88.17	92.47	0.67	0.238	0.030	0.279
Ile	83.41	83.61	83.83	86.1	87.50	90.66	85.85	0.94	89.67	94.74	84.81	90.02	90.63	91.35	90.20	1.57	0.105	0.033	0.355
Leu	86.18	94.26	85.42	88.95	87.86	92.52	89.20	1.11	91.37	95.99	87.42	91.36	91.92	93.00	91.84	1.11	0.059	0.179	0.101
Lys	81.34	89.23	84.96	89.01	88.12	91.30	87.33	3.08	91.40	96.68	86.04	90.58	90.19	93.19	91.35	1.41	0.066	0.078	0.464
Met	87.77	77.12	81.88	84.22	86.26	90.51	84.63	3.80	89.90	90.43	86.71	88.81	88.14	91.16	89.19	1.33	0.658	0.049	0.044
Phe	87.82	88.48	84.67	88.82	88.14	93.19	88.52	1.08	92.55	95.33	87.14	91.82	92.58	93.64	92.18	0.81	0.362	0.044	0.557
Thr	81.82	90.39	78.01	80.32	83.39	84.03	82.99	1.29	83.91	81.83	80.49	83.31	83.84	83.90	82.88	0.79	0.560	0.952	0.434
Trp	85.57	91.70	90.46	90.58	87.57	91.71	89.60	1.91	93.17	92.66	91.46	91.58	91.89	92.66	92.23	0.16	0.100	0.031	0.370
Val	76.54	89.41	85.97	87.15	87.88	91.79	86.46	1.54	88.55	90.71	86.14	90.07	91.05	91.70	89.70	1.48	0.149	0.189	0.171
Dispensable AA																			
Ala	84.24	87.83	79.43	90.56	90.15	90.88	87.18	4.37	89.22	87.96	81.85	91.84	93.22	81.17	87.54	2.10	0.029	0.179	0.609
Asp	85.16	86.56	84.10	88.64	86.88	91.92	87.21	0.99	90.22	91.20	87.01	90.90	90.76	91.65	90.29	0.94	0.088	0.078	0.342
Cys	81.87	90.63	83.28	82.82	90.01	87.83	86.07	3.38	89.83	93.46	86.29	91.88	92.00	89.50	90.49	0.73	0.185	0.042	0.216
Glu	85.77	88.34	86.49	90.50	87.38	91.81	88.38	0.79	90.83	92.54	90.29	91.02	91.29	92.24	91.37	0.73	0.342	0.049	0.378
Gly	86.17	93.38	85.90	87.95	87.00	91.37	88.63	1.08	90.64	92.67	88.67	90.29	90.91	90.61	90.63	0.77	0.084	0.044	0.806
Pro	85.71	89.32	85.55	85.43	86.68	90.57	87.21	0.83	91.34	87.30	88.79	88.29	89.08	90.41	89.20	0.87	0.594	0.952	0.772
Ser	81.11	91.84	82.18	85.28	87.79	89.35	86.26	1.18	87.64	89.25	84.65	87.48	87.79	88.46	87.54	0.99	0.108	0.031	0.551
Tyr	80.36	90.82	78.07	77.02	83.78	83.28	82.22	5.52	88.27	85.44	85.26	85.71	81.82	82.37	84.81	2.15	0.144	0.189	0.435

SIAAD was corrected from AIAAD by eliminating basal ileal endogenous AA loses.

**Table 8:** Standardized amino acid digestibility of different sources BR in broilers (%).

Item	14-DAY-Old								28-Day-Old								*p*-Value (Origin)	*p*-Value (Age)	*p*-Value (Interact)
	BR 1	BR 2	BR 3	BR 4	BR 5	BR 6	Mean	SEM	BR 1	BR 2	BR 3	BR 4	BR 5	BR 6	Mean	SEM			
Indispensable AA																			
Arg	81.17	89.61	76.45	84.28	87.58	87.64	84.45	1.47	82.04	83.00	81.61	87.97	88.56	88.18	85.23	1.00	0.059	0.757	0.638
His	85.16	88.34	85.18	86.92	89.58	85.61	86.80	0.73	90.88	89.60	86.93	90.80	91.51	86.11	89.30	0.67	0.238	0.041	0.369
Ile	80.58	80.79	77.67	83.28	86.34	87.83	82.75	1.30	86.74	91.80	75.21	87.09	87.70	88.42	86.16	1.57	0.105	0.250	0.145
Leu	83.83	81.91	83.07	86.60	88.84	90.17	85.74	0.97	89.17	93.79	81.89	89.16	89.72	90.80	89.09	1.11	0.034	0.042	0.180
Lys	77.60	88.83	77.90	85.27	86.39	87.57	83.93	1.54	84.22	92.84	78.86	86.74	86.35	89.34	86.39	1.41	0.078	0.398	0.157
Met	85.75	78.44	79.86	82.20	84.24	88.49	83.16	1.98	86.47	82.34	81.95	87.38	86.71	89.73	85.76	1.33	0.658	0.216	0.100
Phe	85.70	90.02	85.88	86.70	89.34	91.07	88.12	1.10	90.53	93.31	85.12	89.80	90.56	91.62	90.15	0.81	0.352	0.195	0.298
Thr	73.56	83.46	75.75	76.73	79.79	80.44	78.29	1.31	76.98	78.23	80.23	79.71	80.25	80.30	79.28	0.79	0.560	0.541	0.989
Trp	89.23	88.70	87.46	87.58	87.90	88.70	88.26	0.24	89.67	89.15	87.96	88.08	88.39	89.16	88.73	0.16	0.100	0.270	0.932
Val	73.94	86.80	83.36	84.54	87.61	89.18	84.24	1.57	86.19	89.69	87.12	87.72	88.69	89.34	88.13	0.48	0.149	0.022	0.190
Dispensable AA																			
Ala	74.00	85.92	68.52	86.98	89.91	87.30	82.11	2.22	81.96	82.37	88.59	88.58	89.96	75.58	84.51	2.10	0.004	0.580	0.432
Asp	82.75	84.14	81.68	86.22	87.80	89.50	85.35	0.92	87.94	88.92	81.40	88.62	88.48	89.37	87.45	0.94	0.088	0.254	0.990
Cys	79.02	88.44	80.42	79.96	87.15	84.97	83.33	2.08	85.15	87.45	83.61	89.20	87.52	86.95	86.65	0.73	0.155	0.031	0.097
Glu	84.59	87.16	85.31	89.31	89.53	90.63	87.75	0.62	89.77	91.48	87.57	89.96	90.23	91.18	90.03	0.73	0.312	0.036	0.314
Gly	83.31	93.86	83.04	85.09	87.47	88.51	86.88	1.15	88.16	90.19	82.85	87.80	88.42	88.12	87.59	0.77	0.059	0.722	0.434
Pro	83.29	85.24	83.13	83.01	85.93	88.15	84.79	1.05	88.53	84.49	82.65	85.48	86.27	87.60	85.84	0.87	0.594	0.406	0.253
Ser	78.26	98.90	79.33	82.43	84.94	86.50	85.06	1.82	85.43	87.04	79.11	85.27	85.58	86.24	84.78	0.99	0.108	0.933	0.676
Tyr	73.30	76.78	74.34	69.96	76.72	79.55	75.11	1.10	79.95	70.79	75.61	79.39	78.83	79.38	77.32	1.15	0.144	0.291	0.377

SIAAD was corrected from AIAAD by eliminating basal ileal endogenous AA loses.

**Table 9:** Standardized amino acid digestibility of different sources FFRB in broilers (%).

Item	14-Day-Old								28-Day-Old								*p*-Value (Origin)	*p*-Value (Age)	*p*-Value (Interact)
	FFRB 1	FFRB 2	FFRB 3	FFRB 4	FFRB 5	FFRB 6	Mean	SEM	FFRB 1	FFRB 2	FFRB 3	FFRB 4	FFRB 5	FFRB 6	Mean	SEM			
Indispensable AA																			
Arg	84.09	84.03	83.94	82.20	83.81	77.45	82.59	0.89	90.15	90.02	92.07	88.14	86.44	81.50	88.05	0.91	0.188	0.015	0.074
His	85.33	84.09	82.80	83.85	84.35	80.51	83.49	0.69	90.91	90.10	92.33	91.66	89.25	84.28	89.76	0.90	0.339	0.001	0.239
Ile	81.90	82.24	84.58	83.25	82.36	80.37	82.45	0.61	89.59	89.54	92.44	90.61	87.94	83.15	88.88	0.73	0.112	0.001	0.106
Leu	84.02	79.36	83.83	84.91	77.97	71.53	80.27	1.59	90.26	87.80	91.48	91.17	82.67	79.88	87.21	1.26	0.001	0.037	0.071
Lys	81.73	82.57	85.38	77.93	76.86	74.65	79.85	1.25	90.94	91.42	90.85	79.85	87.96	89.52	88.42	1.01	0.079	0.005	0.083
Met	88.20	86.53	84.94	86.79	86.97	91.10	87.42	0.98	90.76	90.60	88.67	89.06	91.87	92.49	90.58	0.62	0.051	0.013	0.040
Phe	81.77	82.66	83.77	84.55	80.65	88.64	83.67	0.84	89.27	89.44	91.71	91.93	84.22	89.86	89.41	0.67	0.254	0.005	0.357
Thr	83.84	79.70	71.37	79.14	82.90	89.94	81.15	1.51	89.02	84.06	82.07	85.15	87.31	91.75	86.56	0.81	0.037	0.091	0.651
Trp	80.53	83.81	87.99	76.01	67.11	62.40	76.31	2.19	85.73	87.60	93.90	85.73	75.92	76.43	84.22	1.53	0.001	0.139	0.239
Val	79.57	80.25	80.33	78.27	63.08	89.90	78.57	2.90	87.64	86.24	88.54	85.30	72.56	92.99	85.55	2.16	0.001	0.154	0.303
Dispensable AA																			
Ala	79.13	81.06	79.81	84.08	79.98	70.53	79.10	1.26	88.47	88.11	89.08	89.01	83.41	76.54	85.77	1.15	0.032	0.036	0.041
Asp	78.15	77.86	79.42	79.10	81.47	65.46	76.91	1.43	85.41	85.84	86.52	84.97	85.84	69.67	83.04	1.48	0.091	0.116	0.204
Cys	87.09	87.77	75.16	84.98	83.45	66.34	80.80	2.45	88.47	90.48	92.03	90.75	88.47	76.74	87.82	1.35	0.055	0.119	0.274
Glu	86.76	86.91	84.30	83.55	84.65	85.09	85.21	0.60	92.22	92.25	92.67	90.82	89.65	86.07	90.61	0.57	0.299	0.001	0.313
Gly	79.80	79.05	75.92	79.82	80.14	76.73	78.58	0.80	87.52	86.97	85.98	86.51	83.10	77.71	84.63	0.91	0.265	0.005	0.064
Pro	81.70	80.71	81.12	79.48	77.60	66.28	77.82	1.45	89.02	85.68	87.53	87.40	83.31	72.72	84.28	1.37	0.159	0.088	0.682
Ser	78.46	78.73	74.71	77.04	79.21	68.98	76.19	1.14	86.86	87.46	86.52	85.79	83.59	73.75	84.00	1.18	0.180	0.015	0.123
Tyr	81.88	83.55	85.68	79.03	82.52	76.91	81.60	1.02	89.98	90.24	93.44	87.84	87.47	82.32	88.55	1.02	0.098	0.006	0.150

SIAAD was corrected from AIAAD by eliminating basal ileal endogenous AA loses.

**Table 10:** Analyzed standardized amino acid digestibility (SIAAD) of experiment diets of broilers (%).

Item		14-Day-Old Broilers					28-Day-Old Broilers			
	Rice	BR	RB	*p*-Value	SEM	Rice	BR	RB	*p*-Value	SEM
Indispensable AA										
Arg	87.29 ^a^	77.87 ^c^	82.59 ^b^	0.027	1.19	88.75	84.13	88.05	0.744	0.95
His	88.32 ^a^	86.42 ^a^	83.49 ^b^	0.001	0.61	91.80	89.06	89.76	0.244	0.71
Ile	85.85 ^a^	82.29 ^b^	82.45 ^b^	0.011	0.60	90.20	86.87	88.88	0.492	0.79
Leu	89.20 ^a^	82.48 ^b^	80.27 ^b^	0.001	1.25	91.84 ^a^	89.18 ^ab^	87.21 ^b^	0.041	0.92
Lys	87.33 ^a^	79.45 ^b^	79.85 ^b^	0.006	1.25	91.35	87.93	88.42	0.220	0.95
Met	84.63	83.49	87.42	0.161	0.83	89.19	87.31	90.58	0.286	0.58
Phe	88.52 ^a^	84.35 ^b^	83.67 ^b^	0.007	0.80	92.18	89.22	89.41	0.088	0.67
Thr	82.99	78.33	81.15	0.475	1.07	82.88	84.87	86.56	0.069	0.81
Trp	89.60 ^a^	81.65 ^b^	76.31 ^b^	0.002	1.91	92.24 ^a^	83.93 ^b^	84.22 ^b^	0.006	1.33
Val	86.46 ^a^	81.86 ^ab^	78.57 ^b^	0.039	1.55	89.70	87.52	85.55	0.129	1.08
Dispensable AA										
Ala	87.18 ^a^	74.87 ^b^	79.10 ^b^	0.004	1.54	87.54	83.64	85.77	0.475	1.01
Asp	87.21 ^a^	83.56 ^a^	76.91 ^b^	0.001	1.34	90.29 ^a^	87.52 ^ab^	83.04 ^b^	0.011	1.20
Cys	82.22	77.33	81.60	0.773	0.97	84.81	82.43	88.55	0.054	0.92
Glu	88.38 ^a^	85.49 ^b^	85.21 ^b^	0.014	0.56	91.37	88.52	90.61	0.574	0.58
Gly	88.63 ^a^	84.95 ^b^	78.58 ^c^	0.001	1.12	90.63 ^a^	86.95 ^ab^	84.63 ^b^	0.003	0.89
Pro	87.21 ^a^	81.59 ^b^	77.82 ^b^	0.001	1.31	89.20 ^a^	86.51 ^ab^	84.28 ^b^	0.039	0.97
Ser	86.26 ^a^	82.31 ^a^	76.19 ^b^	0.001	1.29	87.55	85.44	84.00	0.083	0.81
Tyr	86.07	82.28	80.80	0.123	1.35	90.49	88.46	87.82	0.238	0.88
Mean	86.85 ^a^	81.70 ^b^	80.66 ^b^	0.001	0.80	89.56	86.64	87.07	0.083	0.60

^a–c^ within a row means those without a common letter are significantly different (*p* < 0.05). SIAAD was corrected from AIAAD by eliminating basal ileal endogenous AA loses

**Table 11:** The linear regression equation of AMEn and SIAAD of selected amino acids.

Item	Liner Regression Equation	R^2^	*p*-Value ^3^
AMEn	Y^2^ = 14.312 − (0.198 × NDF)	0.928	0.001
	Y = 6.491 + (0.103 × Starch)	0.843	0.001
SIAAD			
Thr	X^3^ = 44.65 + (2.151 × GE)	0.317	0.005
	X = 81.07 + (0.067 × NDF)	0.406	0.044
	X = 0.863 + (6.311 × CP)	0.949	0.001
Lys	X = 99.59 − (0.717 × GE)	0.037	0.469
	X = 87.88 − (0.142 × NDF)	0.157	0.103
	X = 91.82 − (0.608 × CP)	0.171	0.088
	X = 62.00 + (0.938STARCH − 0.021 × NDF)	0.863	0.007
Met	X = 56.59 + (1.673 × GE)	0.870	0.016
	X = 85.47 + (0.112 × NDF)	0.183	0.077
	X = 81.46 + (0.578 × CP)	0.889	0.022
Trp	X = 151.99 − (3.585 × GE)	0.295	0.012
	X = 90.84 − (0.364 × NDF)	0.438	0.003
	X = 102.883 − (1.77 × CP)	0.701	0.001

Chemical composition coefficient in liner regression equation represented their concentration (%) in rice, BR, and FFRB. Y^2^ represents AMEn (MJ/kg). X^3^ represents standardized amino acid digestibility (%). *p*-Value ^3^ represents the probabilities of significance for the slopes of the regression equation.

## Data Availability

The data presented in this study are available on request from the corresponding author.

## Supplementary Materials

The following are available online at https://www.mdpi.com/article/10.3390/ani11071894/s1, Table S1: Pearson’s correlation assay between chemical composition of rice and its by-products and AMEn of broiler, Table S2: Basal amino acid losses analysis by NFD method. (%).
