# Supplementary material for: Evaluation of Nitrogen-Corrected Apparent Metabolizable Energy and Standardized Ileal Amino Acid Digestibility of Different Sources of Rice and Rice Milling Byproducts in Broilers
Source: Animals (Basel). 2021 Jun 25;11(7):1894. doi: 10.3390/ani11071894 (PMC8300392; doi:10.3390/ani11071894)
Supplement: Supplementary file 1 [file animals-11-01894-s001.zip › animals-1219965-supplementary.pdf]

**Supplement table 1.** Pearson's correlation assay between chemical composition of rice and its by-products and AMEn of broiler.

| Item | AMEn | DM | CP | EE | NDF[1] | ADF[1] | Starch | Ash | Ca[2] | P[2] |
|---|---|---|---|---|---|---|---|---|---|---|
| AMEn | 1 | | | | | | | | | |
| DM | 0.638** | 1 | | | | | | | | |
| CP | -0.861** | -0.708** | 1 | | | | | | | |
| EE | -0.824** | -0.715** | 0.971** | 1 | | | | | | |
| NDF1 | -0.964** | -0.686** | 0.923** | 0.907** | 1 | | | | | |
| ADF1 | -0.975** | -0.679** | 0.931** | 0.912** | 0.981** | 1 | | | | |
| Starch | 0.918** | 0.722** | -0.985** | -0.963** | -0.954** | -0.970** | 1 | | | |
| Ash | -0.888** | -0.709** | 0.980** | 0.967** | 0.936** | 0.949** | -0.990** | 1 | | |
| Ca | -0.797** | -0.758** | 0.948** | 0.949** | 0.884** | 0.896** | -0.947** | 0.954** | 1 | |
| P | -0.891** | -0.759** | 0.985** | 0.965** | 0.947** | 0.950** | -0.988** | 0.986** | 0.967** | 1 |

* represent significance ($p<0.05$), ** means extremely significant differences ($p<0.01$).

[1]NDF means neutral detergent fiber, ADF means acid detergent fiber.

[2]Ca represent calcium, P means total phosphorus.

**Supplement table 2.** Basal amino acid losses analysis by NFD method. (%)

| Item | 14-day-old | | 28-day-old | |
|---|---|---|---|---|
| | AA losses | SD | AA losses | SD |
| Indispensable AA | | | | |
| Arg | 0.74 | 0.10 | 0.82 | 0.05 |
| His | 0.82 | 0.11 | 0.91 | 0.06 |
| Ile | 0.79 | 0.22 | 0.87 | 0.39 |
| Leu | 0.83 | 0.15 | 0.91 | 0.27 |
| Lys | 0.84 | 0.09 | 0.92 | 0.19 |
| Met | 0.14 | 0.09 | 0.16 | 0.04 |
| Phe | 0.80 | 0.23 | 0.89 | 0.32 |
| Thr | 0.80 | 0.29 | 0.87 | 0.15 |
| Trp | 0.55 | 0.15 | 0.59 | 0.13 |
| Val | 0.79 | 0.11 | 0.86 | 0.14 |
| Dispensable AA | | | | |
| Ala | 0.71 | 1.01 | 0.84 | 0.59 |
| Asp | 0.82 | 0.16 | 0.89 | 0.19 |
| Cys | 0.39 | 0.14 | 0.34 | 0.19 |
| Glu | 0.85 | 0.12 | 0.92 | 0.09 |
| Gly | 0.81 | 0.08 | 0.87 | 0.07 |
| Pro | 0.82 | 0.11 | 0.89 | 0.17 |
| Ser | 0.79 | 0.58 | 0.89 | 0.17 |
| Tyr | 0.51 | 0.17 | 0.57 | 0.20 |

Basal amino acid losses analyzed by NFD method in 14-day-old broilers and 28-day-old broilers.
Data were expressed as mean±SD.
